# How do drought and warming influence survival and wood traits of *Picea mariana* saplings?

**DOI:** 10.1093/jxb/eru431

**Published:** 2014-11-04

**Authors:** Lorena Balducci, Annie Deslauriers, Alessio Giovannelli, Marilène Beaulieu, Sylvain Delzon, Sergio Rossi, Cyrille B. K. Rathgeber

**Affiliations:** ^1^Département des Sciences Fondamentales, Université du Québec à Chicoutimi, 555 boulevard de l’Université, Chicoutimi, QC G7H2B1, Canada; ^2^CNR-IVALSA, Via Madonna de Piano, 50019 Sesto Fiorentino, (FI), Italy; ^3^INRA-University of Bordeaux, UMR BIOGECO, Bat-B2, Avenue des Facultés, 33405Talence-France; ^4^INRA, UMR1092 LERFoB, F-54280 Champenoux, France

**Keywords:** Carbon balance, climate change, drought, survival, warming, wood anatomy, wood density.

## Abstract

Warming and drought will occur with increased frequency and intensity at high latitudes in the future. How heat and water stress can influence tree mortality is incompletely understood. The aim of this study was to evaluate how carbon resources, stem hydraulics, and wood anatomy and density determine the ability of black spruce saplings to survive daytime or night-time warming (+ 6 °C in comparison with control) in combination with a drought period. Plant water relations, the dynamics of non-structural carbohydrates and starch, mortality rate, and wood anatomy and density of saplings were monitored. Warming, in conjunction with 25 d of water deficit, increased sapling mortality (10% and 20% in night-time and daytime warming, respectively) compared with the control conditions (0.8%). Drought substantially decreased gas exchange, and also pre-dawn and mid-day leaf water potential to values close to –3MPa which probably induced xylem embolism (xylem air entry point, *P*_12_, being on average around –3MPa for this species). In addition, the recovery of gas exchange never reached the initial pre-stress levels, suggesting a possible loss of xylem hydraulic conductivity associated with cavitation. Consequently, mortality may be due to xylem hydraulic failure. Warmer temperatures limited the replenishment of starch reserves after their seasonal minimum. Lighter wood was formed during the drought period, reflecting a lower carbon allocation to cell wall formation, preventing the adaptation of the hydraulic system to drought. Saplings of black spruce experienced difficulty in adapting under climate change conditions, which might compromise their survival in the future.

## Highlight

Night and day warming combined with drought affect wood anatomy and survival, reflecting the importance of carbon–water relations for the survival process in *Picea mariana* saplings.

## Introduction

In boreal ecosystems, an increase in temperature of ~2–4 °C is expected by 2060 due to global warming (Plummer *et al.*, 2006; IPCC, 2013). The projected change in climate extremes could have important consequences on tree growth and survival. Recent increases in tree mortality were assumed to be caused by drought and heat stress associated with global change (Breshears *et al.*, 2005; Allen *et al.*, 2010), with repercussions at higher latitudes (Hogg and Bernier, 2005). In the last decades, Peng *et al.* (2011) found that regional drought increased tree mortality in mature stands of the Canadian boreal forest. Drought conditions particularly affect growth and survival of stand regeneration (Payette and Filion, 1985; Hogg and Schwarz, 1997), because young trees are more vulnerable to root embolism and stomatal closure (Domec *et al.*, 2004; Mueller *et al.*, 2005). The increases in temperature occurring during drought also lead to a more rapid dehydration of young trees because of a higher evapotranspiration demand (Angert *et al.*, 2005).

Recent research has emphasized the importance of carbon storage in the physiological response of trees under global environmental changes, such as the increase of CO_2_ (Körner, 2003), temperature (Adams *et al.*, 2009), or drought (McDowell *et al.*, 2008). Tree mortality due to drought is a critical component in North-American boreal forests (Peng *et al.*, 2011). Three main hypotheses have been proposed concerning the physiological mechanisms leading to tree mortality during drought: (i) the carbon starvation hypothesis, suggesting that a prolonged stomatal closure leads to a depletion of carbohydrate reserves (McDowell *et al.*, 2008); (ii) the hydraulic failure hypothesis, reflecting a strong alteration of water transport (Brodribb *et al.*, 2010; Urli *et al.*, 2013); and (3) biotic disturbance, indicating that pathogens and insects amplify the effects of the two previous mechanisms (McDowell *et al.*, 2008). Recurrent droughts could affect plant carbon balance, while severe droughts result in xylem embolism, both leading to increased mortality in forest stands (McDowell *et al.*, 2008). However, an intriguing debate on the occurrence of carbon starvation (Leuzinger *et al.*, 2009; Sala, 2009; Sala *et al.*, 2010) or a possible coupling of the first two hypotheses (McDowell, 2011) suggests that more studies are needed to elucidate the complex mechanisms involved in tree survival under environmental constraints.

Temperatures are not expected to change equally during the night and day: between 1950 and 1993, the night-time air temperature increased at about twice the rate of maximum air temperature (IPCC, 2001). This tendency was confirmed for the boreal forest in Canada (Bonsal *et al.*, 2001, 2011). Nocturnal warming has several impacts on physiological mechanisms, such as carbon storage and water relations (Sage, 2002; Turnbull *et al.*, 2002). Night-time water uptake and stem sap flow depend on the availability of water in the soil and on the previous day’s plant transpiration (Fuentes *et al.*, 2013). High night-time temperature limits the plant’s water recovery, impairing the water balance of the next day, leading to higher stem water shortage, especially during summer drought, when the nights are shorter (Zeppel *et al.*, 2012; Fuentes *et al.*, 2013). During fatal water status, when the plant does not recover from water stress, water transport is reduced by low stem hydraulic conductivity due to the presence of embolisms (Brodribb *et al.*, 2010). High night-time temperature also alters the carbon balance: nocturnal warming increases maintenance respiration (Turnbull *et al.*, 2002, 2004), leading to a faster degradation of the transitory starch in the chloroplast and thus decreasing the carbon intended to support respiration and growth at night and during the following day.

Wood density is highly sensitive to environmental conditions (Rozenberg *et al.*, 2002; Bouriaud *et al.*, 2005), especially to low or high temperatures. During the growing season, an early temperature decline can induce a reduction in lignin content within terminal tracheids of the latewood (LW) (Gindl *et al.*, 2000), showing a marked effect of temperature on the lignification processes. In black spruce, the inhibition of LW formation can lead to the development of a light ring, due to a shortened growing season and low temperature caused by volcanic eruptions (Filion *et al.*, 1986). In a recent study, it was shown that wood density was also susceptible to warmer conditions (Balducci *et al.*, 2013). Because wood density mainly depends on cell dimensions and the quantity of structural carbon (cellulose, hemicelluloses, and lignin) forming the secondary cell wall (Gindl *et al.*, 2000; Emiliani *et al.*, 2011) the variations in wood density are reflected in the hydraulic architecture of plants. According to the Hagen–Poiseulle law, cells with a higher lumen area are more efficient in water transport than smaller tracheid elements with thicker cell walls, but less resistant to embolism at high (less negative) leaf water potential (Domec and Gartiner, 2002). Wood density is the final balance of carbon investment during wood formation (soluble carbon converted into structural carbon) and is a key factor in defining the final proportion between the cell wall and lumen area (Chave *et al.*, 2006; Rathgeber *et al.*, 2006; Dalla-Salda *et al.*, 2011). Consequently, wood density could help to explain the efficiency versus safety of the xylem. A new challenge could therefore be to understand how sugars and wood density may influence cavitation and survival in response to temperature and drought.

The aim of this study was to evaluate how combined water deficit and temperature increase affected the dynamics of water, sugar, and starch in the stem, the resulting wood anatomy and density, and sapling survival in black spruce. The hypothesis was tested that water deficit coupled with increased night and day temperatures can alter the water and carbon balance of the plant, which results in (i) a reduction in carbon storage in the stem during the night and an increase during the day; (ii) an altered wood anatomy and density (i.e. an increased structural carbon investment); and (iii) exacerbated sapling mortality.

## Materials and methods

### Experimental design

The experiment was conducted during the 2011 growing season in Chicoutimi, Canada (48°25′N, 71°04′W, 150 m above sea level) on 4-year-old black spruce saplings [*Picea mariana* (Mill.) B.S.P.]. In summer 2010, before the beginning of the experiment, the saplings were transplanted into plastic reversed-conic pots (4.5 litres in volume) and grown in an open field until the following spring. In April 2011, a total of 1104 saplings of homogeneous size (53.01±8.8cm in height and 10.43±1.79mm in diameter at the collar) were randomly selected and fertilized with 1g l^–1^NPK (20-20-20) dissolved in 500ml of water to avoid nutrient deficiency. The saplings were arranged in three adjacent greenhouses, where they were grown until October 2011. During the experiment, sapling growth (368 saplings per treatment) was investigated under three different thermal conditions: control (named T0), corresponding to external air temperature; and two warming conditions (T+Day and T+Night), which were 6 °C warmer than T0 during the day (from 07.00h to 19.00h) and during the night (from 19.00h to 07.00h), respectively. In addition, during maximum xylem growth, when saplings are more susceptible to dry conditions (Rossi *et al.*, 2006), two irrigation regimes were applied: (i) control (named, irrigated saplings), consisting of maintaining the soil water content at ~80% of field capacity; and (ii) water deficit (named, non-irrigated saplings), in which irrigation was withheld for 25 d in June [from day of the year (DOY) 158 to 182] in 184 saplings per thermal condition.

### Water relations, gas exchange, and CO_2_ assimilation

Water relations, gas exchange, and CO_2_ assimilation were measured from May to August on branches of the first whorl of 18 saplings per week (3 saplings×3 thermal conditions×2 irrigation regimes) (Supplementary Fig. S1 available at *JXB* online). In each sapling, pre-dawn [Ψ_pd_] and mid-day [Ψ_md_] leaf water potential were measured using a pressure chamber (PMS Instruments, Corvalis, OR, USA). The minimum leaf water potential [Ψ_min_] was considered as the daily minimum pre-dawn and mid-day water potential, [Ψ_min pd_] and [Ψ_min md_], respectively. They were recorded for each irrigation regime under different thermal conditions (Meinzer *et al.*, 2009). In each plant, gas exchange (stomatal conductance, *g*_s_, mol m^–2^ s^–1^) and CO_2_ assimilation (maximum photosynthesis rate, *A*_max_, μmol m^–2^ s^–1^) were measured from 10.00h to 13.00h under saturating irradiance conditions (1000 μmol m^–2^ s^–1^) using a portable photosynthesis system (Li-6400, LI-COR Inc., Lincoln, NE, USA). Air temperature, vapour pressure deficit, CO_2_ concentration, and irradiance inside the chamber were maintained at 25 °C, 2.2±0.7 kPa, 400 μmol mol^–1^ and 1000 μmol m^–2^ s^–1^ photosynthetic photon flux density (PPFD), respectively. Measurements were expressed according to the specific needle surface area computed as the ratio of needle dry mass per unit of needle surface area and using a regression according to Bernier *et al.* (2001). For the same plants, the volumetric water content (VWC) of the soil was measured weekly by time domain reflectometry (TDR Fieldscout 300). The measurements were taken at 7cm depth in each pot and replicated twice (Topp *et al.*, 1984).

Sapling stems of homogeneous size (49.5±8.3cm in height and 6.7±0.8mm in diameter at the collar), straight and without needles, were selected. They were collected in the early morning to minimize xylem tension. The centrifuge method was used to measure the vulnerability of branch xylem to water stress-induced cavitation caused by air seeding (Delzon *et al.*, 2010). The xylem hydraulic conductivity (*k*_s_; m^2^ MPa^–1^ s^–1^), embolism vulnerability (*P*_12_, xylem air entry point; MPa), *P*_50_, pressure inducing 50% loss of hydraulic conductance; MPa), and slope of the vulnerability curve (*S*; % MPa^–1^) of the stem were measured using the cavitron technique on five control saplings (Cochard *et al.*, 2005; Delzon *et al.*, 2010). The centrifugation-based technique was used to establish negative pressure in the xylem and to provoke water stress-induced cavitation, using a custom-built honeycomb rotor (Precis 2000, Bordeaux, France) mounted on a high-speed centrifuge (Sorvall RC5, Asheville, NC, USA) (Delzon *et al.*, 2010). The difference between [Ψ_min_] and the xylem pressures at which PLC=50% (Ψ50) was calculated; this corresponds to a safety margin for the saplings (Meinzer *et al.*, 2009; Choat *et al.*, 2012). For irrigated saplings, the predicted native embolism (PLC_p_) was estimated from minimum mid-day water potential [Ψ_min_] and the vulnerability curve (VC) (Delzon *et al.*, 2010; Urli *et al.*, 2013).

### Mortality

Sapling mortality was monitored weekly from May to October and assessed according to the total number of experimental plants (1104) (Supplementary Fig. S1 at *JXB* online). Every week, the mortality percentage was calculated from the total number of saplings that had died per irrigation regime and thermal condition, excluding saplings randomly selected every week from each treatment for the analysis of total non-structural carbohydrates (NSCs) and starch. Sapling mortality was determined by complete needle wilting and stem necrosis.

### Wood anatomy and density

Stem discs of 36 saplings (6 saplings×3 thermal conditions×2 irrigation regimes) were randomly collected during the last 3 weeks of October (Supplementary Fig. S1 at *JXB* online). Wood sections were stained with safranine (1% in water) and fixed on slides with histological mounting medium. Digital images were recorded using a camera mounted on a microscope to measure xylem features along three paths using WinCell™ (Regent Instruments Inc., Canada). For each cell along the paths, lumen area, radial diameter, and wall thickness were measured. For each anatomical section, earlywood (EW) and LW were identified according to Mork’s formula, which classifies all cells with a lumen smaller than twice a double cell wall as LW (Denne, 1988). The stem discs were air-dried until 12% moisture content and X-rayed together with a calibration wedge following standard techniques (Polge and Nicholls, 1972). Radiographs were digitalized using a scanner, and the acquired digital images were treated using semiautomatic procedures in order to produce tree-ring microdensity profiles (Mothe *et al.*, 1998). Each tree ring was divided into 10 equal parts size considering the relative percentage distance from the beginning of the ring (Mothe *et al.*, 1998).

### Analysis of non-structural carbohydrates and starch

The cambium and xylem tissues of 18 saplings (3 saplings×3 thermal conditions×2 irrigation regimes) were collected every 2 weeks (Supplementary Fig. S1 at *JXB* online) and NSCs were extracted following the procedure described in Giovannelli *et al.* (2011). An Agilent 1200 series HPLC with a RID and a Shodex SC 1011 column and guard column, equipped with an Agilent Chemstation for the LC systems program, was used for assessment of soluble carbohydrates. A calibration curve was created for each carbohydrate using standard sucrose, raffinose, glucose, fructose (Canadian Life Science), and d-pinitol (Sigma-Aldrich). Total NSCs were calculated as the sum of soluble carbohydrate concentrations (raffinose, sucrose, glucose, pinitol, and fructose). Xylem powder was also used for starch extraction, performed according to Chow and Landhäusser (2004). The starch was solubilized with 0.1M NaOH and 0.1M acetic acid, and was hydrolysed enzymatically with an α-amylase solution at 2000U ml^–1^ and amyloglucosidase at 10U ml^–1^. PGO-colour reagent and 75% H_2_SO_4_ were added to the solution 24h later. Starch was assessed using a spectrophotometer at 533nm (Chow and Landhäusser, 2004).

### Statistical analyses

The means of xylem anatomy and density were compared using two-way analysis of variance (ANOVA) with Tukey’s test (*P*<0.05). The comparisons among thermal conditions were performed using the slice option procedure in SAS (SAS Institute, Cary, NC, USA). For each sample, a sigmoid function (Pammenter and Willigen, 1998; Urli *et al.*, 2013) was fitted to the vulnerability curve using proc NLIN in SAS according to the equation:

$$PLC=\frac{100}{1+\exp\left(\frac{S}{25(Pi-P50)}\right)}$$

where *P*_50_ is the pressure inducing 50% loss of hydraulic conductance (MPa) and *S* is the slope of the vulnerability curve (% MPa^–1^) of the stem at the inflection point (Urli *et al.*, 2013). For each thermal condition, the total soluble sugars in cambium and xylem were compared, for each day, between irrigation regimes by using Wilcoxon non-parametric analysis (*P*=0.05). Wilcoxon non-parametric starch comparisons were conducted using the NPAR1WAY procedure in SAS. However, due to a very low number of samples on some days of the year (*n*<3), some tests could not be performed.

## Results

### Growth conditions

**Fig. 1.**
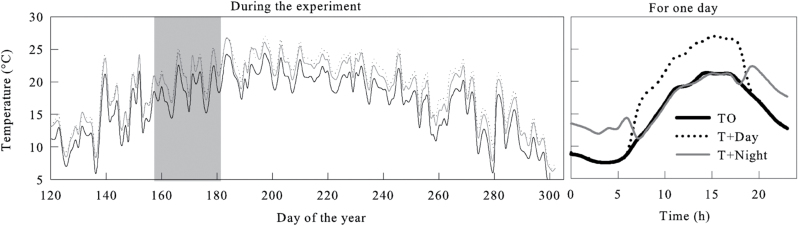
Daily temperatures experienced by black spruce saplings in the three thermal conditions (T0, control temperature; T+Day, temperature increase during the day; T+Night, temperature increase during the night) during the greenhouse experiment from April to October. Grey background corresponds to the water deficit period during June.

During the experiment, mean T+Day and T+Night temperatures were, on average, 4.5 °C and 5.2 °C warmer than T0, as heating was applied from 07.00h to 19.00h in T+Day and from 19.00h to 07.00h in T+Night (Fig. 1). During the water deficit period, the temperature in T0 varied between 14 °C and 22 °C. Maximum temperatures of ~24 °C were reached in July for T0. A gradual decrease in temperature was then observed from the end of August, with a minimum of 3.8 °C in October (Fig. 1).

**Fig. 2.**
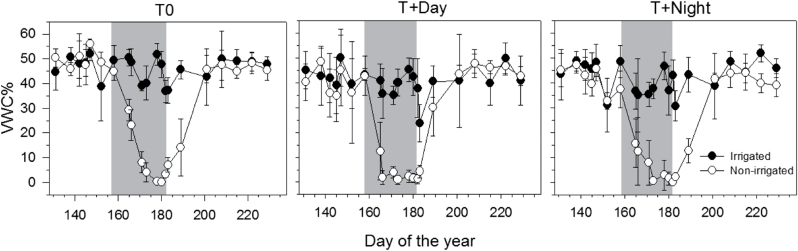
Volumetric water content (VWC) of soil in irrigated saplings (filled circles) and non-irrigated saplings (open circles) before, during, and after the water deficit period (grey background) under the three thermal conditions (T0, control temperature; T+Day, temperature increase during the day; T+Night, temperature increase during the night) during the greenhouse experiment in 2011. Vertical bars represent the standard deviation.

During the period of water deficit, the VWC of non-irrigated saplings decreased in all thermal conditions. After the drought period, VWC increased quickly and field capacity was reached on DOY 200, 20 d after the resumption of irrigation (Fig. 2). After DOY 183, the VWC was maintained at field capacity until the end of the experiment.

### Sapling mortality

The treatments generated a high mortality rate of saplings, especially after the drought period (during June). In the irrigated treatments, all trees survived under the three thermal conditions (Table 1). In the non-irrigated saplings, the rate of mortality increased proportionally with higher night-time and daytime temperatures. One week after the end of water deficit, the mortality was 0.8% in T0, 10.48% in T+Night, and 19.55% in T+Day. At the end of July (DOY 202, 3 weeks after re-watering), mortality persisted in all thermal conditions but was much lower in T+Day and T0, with values of 0.44% and 0.8%, respectively. Higher sapling mortality was still observed in T+Night, with the value reaching 1.78% (Table 1).

**Table 1.** Percentage of mortality 1 and 3 weeks after the water deficit period (WDp)

	% Sapling mortality		
	T0	T+Day	T+Night
Irrigated			
1 week after WDp	0	0	0
3 weeks after WDp	0	0	0
Non-irrigated			
1 week after WDp	0.81	19.76	10.48
3 weeks after WDp	0.89	0.45	1.79

### Sapling water relations, gas exchange, and CO_2_ assimilation

**Fig. 3.**
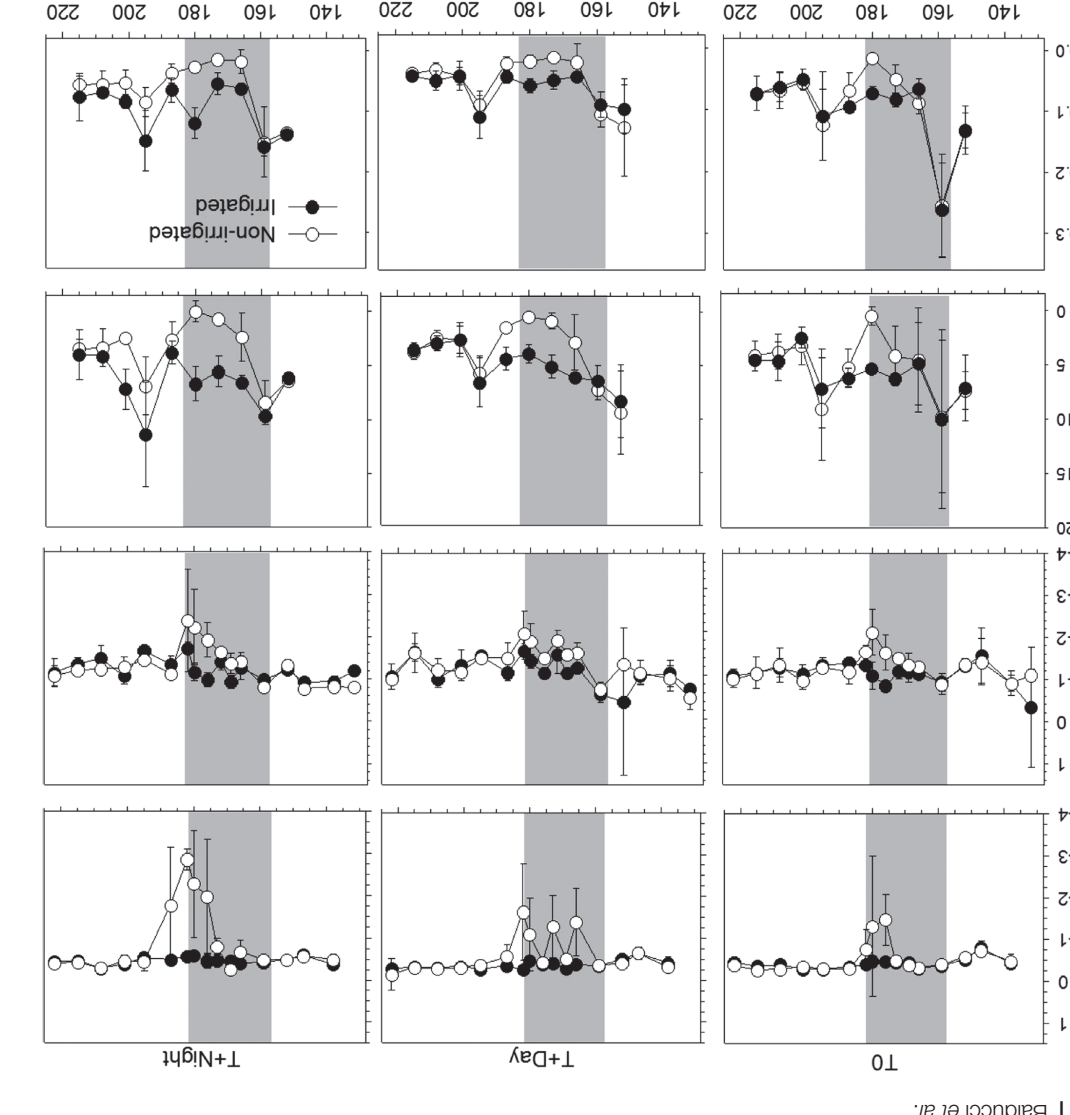
Pre-dawn leaf water potential (Ψ_pd;_ MPa), midday leaf water potential (Ψ_md;_ MPa), CO_2_ assimilation (maximum photosynthesis rate, *A*_max_; μmol m^–2^ s^–1^), and gas exchange (stomatal conductance, *g*_s_; mol m^–2^ s^–1^) of black spruce saplings before, during, and after the water deficit period (WDp) under the three thermal conditions during the greenhouse experiment in 2011.

Similar patterns of gas exchange were observed in the irrigated regimes, with a small increase of *A*_max_ at higher daytime temperature: the value of *A*_max_ was 7 μmol CO_2_ m^–2^ s^–1^ for T0 compared with 8 and 9 μmol CO_2_ m^–2^ s^–1^ in T+Day (Fig. 3). During the drought period, *A*_max_ ranged between 4 and –0.04 μmol CO_2_ m^–2^ s^–1^ in non-irrigated saplings. A faster decrease was observed in T+Day and T+Night, starting on DOY 166 until the end of the drought period. Similar patterns were observed for *g*_s_, which was lower than 0.02 (values) in T0 under high temperature (DOY 166) (Fig. 3).

Overall, the recovery of gas exchange after rewatering was much slower at higher daytime and night-time temperatures. Indeed, the gas exchange values of non-irrigated saplings were similar to those of irrigated saplings after 1 week in T0, 2 weeks in T+Day, and 4 weeks in T+Night. In addition, gas exchange never recovered to pre-stress levels in both temperature treatments, probably due to the relatively slow recovery of xylem hydraulic conductivity following rewatering.

In April and May, pre-dawn and mid-day leaf water potential showed optimal water status for all treatments (Fig. 3). During the period when irrigation was withheld, pre-dawn leaf water potential (Ψ_pd_) gradually dropped, with a more pronounced decrease at higher night-time temperature. The Ψ_pd_ values of irrigated saplings were close to zero (ranging between −0.3 and −0.4±0.1MPa), demonstrating an optimal plant water status. In non-irrigated saplings, Ψ_pd_ gradually dropped with increasing daytime and night-time temperature, with values of − 0.7±0.4MPa for T0, −1.63±1.1MPa for T+Day, and −2.8±0.2MPa for T+Night. From DOY 171 to 181, lower values of Ψ_md_ were observed in non-irrigated saplings, with values reaching –2.1±0.5MPa in T0, –1.95±0.5MPa, and –2.38±0.1MPa in T+Day and T+Night, respectively. The minimum leaf water potential values (Ψ_min md_) were also low, with values of −2.1MPa and −1.97MPa in T0 and T+Day, respectively, and the lowest values observed in T+Night (Ψ_min pd_, −2.8MPa). After resumption of irrigation, the recovery of plant water status differed between the thermal conditions. The leaf water potential did not differ between the irrigated and non-irrigated saplings at T0. However, saplings growing at T+Day and T+Night showed a slower plant water status recovery, with a delay of 2 and 4 weeks, respectively. The non-irrigated saplings needed 1 week for the recovery of mid-day leaf water potential under all thermal conditions.

**Fig. 4.**
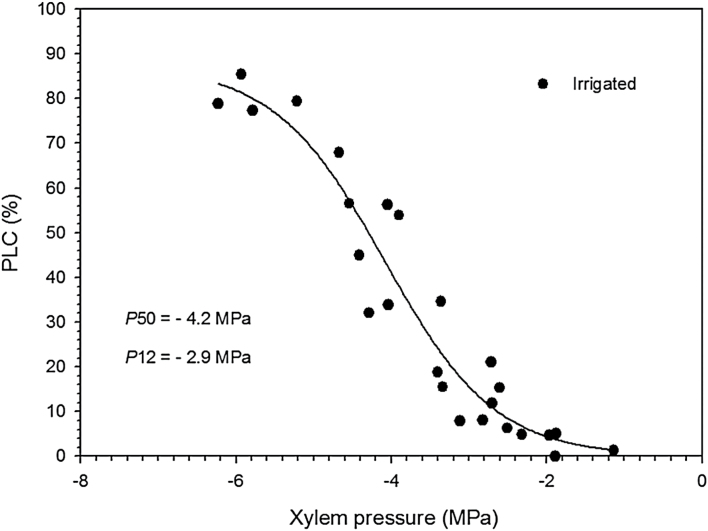
Mean percentage loss of hydraulic conductance (PLC%) versus xylem pressure (MPa) for black spruce saplings The vulnerability curve was obtained with the cavitron technique.

The xylem pressure inducing 50% loss of conductance (*P*_50_) reached average values of −4.26MPa, and the air point entry (*P*_12_) reached average values of −2.95MPa in irrigated saplings (Fig. 4; Table 2). The slope of the vulnerability curve (*S*) was 41.71% MPa^–1^ (Table 2). The difference between Ψ_min_ and Ψ_50_ was 2.59MPa for irrigated saplings. The values of PLC_p_ ranged from 4.8% to 8.0%.

**Table 2.** Mean values and SD of xylem pressure inducing 50% loss in conductance (P_50_), xylem air entry point (P_12_), and vulnerability curve slope of the stem measured on black spruce saplings

Parameters	*Picea mariana* sapling
	Irrigated
*P*_50_ (MPa)	–4.27±0.1
*P*_12_ (MPa)	–2.95±0.05
Slope (% MPa^–1^)	41.72±16.70

### Wood anatomy and density

**Fig. 5.**
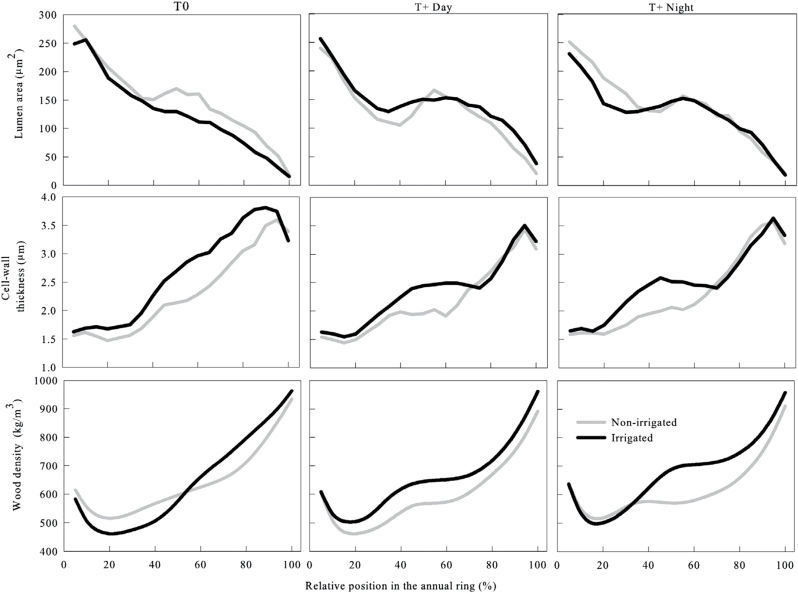
Cell features and wood density of the tracheids produced by irrigated (black curves) and non-irrigated (grey curves) black spruce saplings under three thermal conditions (T0, control temperature; T+Day, temperature increase during the day; T+Night, temperature increase during the night) along portions of an annual tree ring.

Both treatments affected cell features and wood density. Along the tree rings, cell lumen area of T0 progressively decreased from ~ 300 μm^2^ to 20 μm^2^ (Fig. 5). Under warmer conditions, statistical differences between treatments were observed; the cell lumen remained stable in the central portion of the tree ring with values ranging from 150 μm^2^ to 100 μm^2^, and then it decreased to minimal values of 20–29 μm^2^ under high temperature conditions at the end of the annual ring.

Cell wall thickness was affected by water deficit under all thermal conditions. In the first 20% of the ring, cell wall thicknesses were quite similar between all treatments (with values ranging between 1.5 μm and 1.7 μm; Fig. 5). However, statistically significant differences were observed between irrigation regimes in all thermal treatments (Table 4). In non-irrigated saplings, the cell wall thickness of the cells located in 50–85% of the tree ring remained at ~2 μm (Fig. 5). In comparison, the cell wall thickness of irrigated saplings kept increasing and reached values of ~2.5–3 μm in T+Day and T+Night. The maximum cell wall thickness was reached in LW, with values of 3.6 μm at 90% of the tree ring for non-irrigated saplings and 4 μm for irrigated saplings at 85–95% of the tree ring.

Wood density was affected by water deficit and night-time temperature. Wood density profiles increased along the annual tree ring, with higher values found in LW (Fig. 5). The average values of wood density in the different thermal conditions were 643, 630, and 648.70kg m^–3^ at T0, T+Day, and T+Night, respectively (Table 3). At 50–85% portions of the annual ring, a significant difference was observed between the irrigation regimes, with irrigated saplings showing higher density values than non-irrigated saplings (*P*<0.05) (Table 4). Under the warming conditions, significant differences were observed at T+Night (*P*<0.0001). In irrigated saplings, the values of maximum wood density were similar under all thermal conditions, ranging from 982kg m^–3^ to 991kg m^–3^. A progressive decrease of maximum wood density under high thermal conditions was observed in the non-irrigated saplings (Table 3). In irrigated saplings, the minimum wood density increased with warming, while in non-irrigated saplings the density decreased with warming (Table 3). Fluctuations in wood density were recorded in the 50–85% portions of the annual ring especially at increased night-time temperatures, with irrigated saplings showing higher values than non-irrigated saplings (Fig. 5; Table 4).

**Table 3.** Wood properties (mean and SD) of black spruce saplings at three thermal conditions during the greenhouse experiment in 2011)

	Irrigation regimes	T0	T+Day	T+Night
Wood density (kg m^–3^)				
Mean	Non-irrigated	638±86	598±79	621±99
	Irrigated	650±79	657±79	676±95
Minimum	Non-irrigated	481±90	435±61	470±73
	Irrigated	438±59	478±86	478±96
Maximum	Non-irrigated	954±153	917±141	936±145
	Irrigated	991±120	985±109	982±100
Earlywood	Non-irrigated	566±87	541±60	577±88
	Irrigated	540±52	598±80	590±99
Latewood	Non-irrigated	832±125	785±146	811±121
	Irrigated	850±108	844±104	825±99
Ring width (mm)				
Earlywood	Non-irrigated	0.56±0.2	**0.59±0.2**	0.71±0.3
	Irrigated	0.64±0.2	**0.81±0.2**	0.59±0.2
Latewood	Non-irrigated	0.22±0.2	0.23±0.2	**0.15±0.1**
	Irrigated	0.33±0.1	0.28±0.2	**0.33±0.2**
Proportion (%)				
%Earlywood	Non-irrigated	71.8	73.6	70.8
	Irrigated	60.5	74.0	68.8
%Latewood	Non-irrigated	28.2	26.4	29.2
	Irrigated	39.5	26.0	31.2

Significant effects between irrigation regimes (*P*≤0.05) are in bold.

**Table 4.** *P* values for wood density, cell-wall thickness, and lumen area along relative portion of tree ring (%) in black spruce saplings calculated between irrigation regimes (I), among thermal conditions (T) and interaction between irrigation regimes and thermal conditions (I × T)

Relative portion of tree ring (%)	Lumen area			Cell-wall thickness			Wood density		
	I	T	I × T	I	T	I × T	I	T	I × T
5	**0.0734**	**0.0125**	**0.0086**	**0.0180**	0.6222	0.9311	0.863	0.2488	0.9572
10	0.1833	**<.0001**	**0.0368**	**0.0201**	**0.0227**	0.9393	0.6981	0.569	0.5622
15	**0.0374**	**<.0001**	**0.0005**	**0.0041**	**0.0012**	0.2776	0.7275	0.6738	0.3188
20	**<.0001**	**<.0001**	**<.0001**	**0.0003**	**0.0005**	0.2431	0.7871	0.5327	0.2653
25	**0.0370**	**<.0001**	**<.0001**	**<.0001**	**0.0044**	0.1173	0.8825	0.3263	0.224
30	**0.0162**	**<.0001**	**<.0001**	**<.0001**	**<.0001**	**0.0130**	0.8295	0.1777	0.1457
35	0.6011	**<.0001**	**0.0103**	**<.0001**	**<.0001**	**0.0152**	0.5039	0.1032	0.1128
40	0.1043	**0.0002**	**<.0001**	**<.0001**	**0.0065**	**0.0155**	0.2266	0.0798	0.1274
45	0.8612	**0.0193**	**<.0001**	**<.0001**	**0.0022**	0.1574	0.0641	0.131	0.1314
50	**0.0132**	0.5606	**<.0001**	**<.0001**	**<.0001**	0.5906	**0.0148**	0.3645	0.1224
55	**<.0001**	**0.0007**	**0.0021**	**<.0001**	**<.0001**	**0.0151**	**0.0048**	0.666	0.1765
60	**<.0001**	**0.0008**	**<.0001**	**<.0001**	**<.0001**	**0.0005**	**0.0035**	0.5406	0.3595
65	**0.0076**	**<.0001**	**0.0119**	**<.0001**	**<.0001**	**<.0001**	**0.0038**	0.3249	0.6323
70	0.0761	**<.0001**	**<.0001**	**<.0001**	**<.0001**	**<.0001**	**0.0053**	0.2262	0.7925
75	0.118	**<.0001**	**<.0001**	**0.0202**	**<.0001**	**<.0001**	**0.0076**	0.1696	0.7991
80	0.1289	**<.0001**	**<.0001**	**0.0247**	**<.0001**	**<.0001**	**0.0134**	0.1391	0.8223
85	0.9564	**<.0001**	**<.0001**	**0.0157**	**<.0001**	**<.0001**	**0.0281**	0.1453	0.924
90	**0.0207**	**<.0001**	**<.0001**	0.0643	**<.0001**	**0.0018**	0.0569	0.2142	0.9931
95	0.6998	**<.0001**	**<.0001**	**0.0453**	**0.0033**	0.7037	0.0987	0.4413	0.9516
100	**0.0268**	**<.0001**	**<.0001**	0.3345	**0.0076**	**0.0023**	0.0987	0.8289	0.8944

Significant effects (*P*≤0.05) are in bold.

In irrigated saplings, EW represented ~60% of the tree ring at T0, while the proportion increased to ~74% and 68% at T+Day and T+Night, respectively. In non-irrigated saplings, the EW values ranged between 70% and 73%. Consequently the proportion of LW was greater in irrigated saplings at T0. On average, the EW width of the irrigated saplings increased at T+Day and, in non-irrigated saplings, increased at T+Night (Table 3). Statistical differences were found in EW width at T+Day and in LW width at T+Night (Tables 3, 4).

### Dynamics of total NSCs and starch

**Fig. 6.**
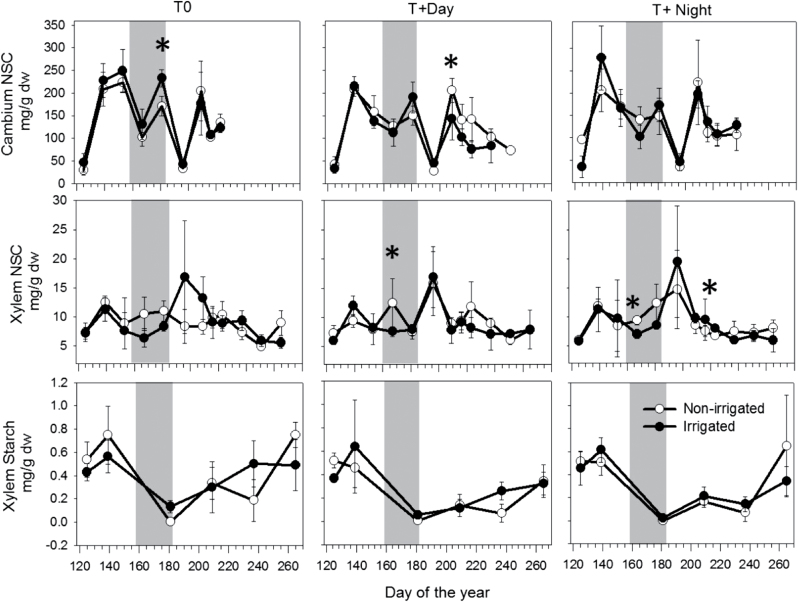
Non-structural carbohydrates (NSCs) in mg g_dw_^–1^ in cambium and in xylem, and starch concentration in xylem (mg g_dw_^–1^) in black spruce saplings before, during, and after the water deficit period (grey background) under three thermal conditions (T0, control temperature; T+Day, 6 °C higher daytime temperature; T+Night, 6 °C higher night-time temperature). Filled and open white circles indicate the two irrigation regimes. Asterisks indicate statistically significant differences between the two irrigation regimes (Wilcoxon test; *P*≤0.05).

During the growing season, similar concentrations of total NSC were observed in the cambium under all thermal conditions (Fig. 6). At the beginning of the experiment (DOY 125), average values of total NSCs ranged from 40mg g^–1^ to 50mg g^–1^ in irrigated saplings. In non-irrigated saplings, the value was 34mg g^–1^ at T0, whereas it was 49mg g^–1^ and 97mg g^–1^ at T+Day and T+Night, respectively. From DOY 139 to DOY 181, an increase was observed in the total NSCs, with values ranging from 100mg g^–1^ to 200mg g^–1^ in all treatments. Two weeks after re-watering (DOY 196), a synchronous and drastic drop in NSCs was found in all treatments. On DOY 209, the total NSCs in cambium were again high, with mean values ranging from 150mg g^–1^ to 200mg g^–1^, and then decreased gradually at the end of September (Fig. 6). In the xylem, total NSCs changed in a similar manner during the growing season in all treatments (Fig. 6). Similar concentrations were observed at the beginning and end of the experiment, with higher amounts observed on DOY 195 for all thermal conditions, except in non-irrigated saplings for T0.

At the beginning of the experiment, similar amounts of starch were observed in the irrigation regimes (Fig. 6). Starch reached its maximum values on DOY 118 and 139 (ranging from 4mg g^–1^ to 6mg g^–1^), and dropped to zero at the end of water deficit (DOY 181) under all thermal conditions (Fig. 6). After re-watering, starch showed a similar pattern among the irrigation regimes, ranging from 0.24mg g^–1^ to 0.23mg g^–1^. However, a slower increase was observed after the summer minimum under warmer conditions, with a lower concentration in T+Day (0.47mg g^–1^) and T+Night (0.48mg g^–1^) compared with T0 (0.62mg g^–1^). A significant difference was found in the starch concentration between temperature treatments, whereas no difference was found between irrigation regimes and their interaction (Supplementary Table S1 at *JXB* online).

## Discussion

### Mechanisms of sapling survival

An increase in air temperature in conjunction with 25 d of water deficit induced a significant increase in sapling mortality (~10% in T+Night and 20% in T+Day). Mortality persisted as long as 3 weeks after the resumption of irrigation, especially at higher night-time temperature. The observations were in agreement with the mortality observed in 3-year-old black spruce seedlings on regenerated cutover (Ruel *et al.*, 1995). Previous research showed that juvenile mortality in black spruce ranged from 10% to 21% according to stem height and the presence of stem wounds, as the root system cannot withstand drought (Ruel *et al.*, 1995). Another study observed that warmer temperatures during growth triggered a higher percentage of mortality in black spruce seedlings with consequent growth compensation (Way and Sage, 2008b). During the water deficit, stomatal conductance and CO_2_ assimilation in non-irrigated saplings were lower compared with irrigated saplings at high temperature, as found by Way and Sage (2008a).

During water stress, stomatal conductance was strongly reduced when leaf Ψ_pd_ ranged from –1.0MPa to –1.5MPa (Bernier, 1993; Stewart *et al.*, 1994), and damage to the root system of black spruce was observed when Ψ_pd_ reached –2.5MPa (Johnsen and Major, 1999). The results showed that a higher percentage of mortality occurred in T+Day when Ψ_pd_ reached a level of –1.6MPa and Ψ_min md_ was –1.97MPa. The mortality was lower in T+Night, even when Ψ_min pd_ reached –2.8MPa and Ψ_md_ –2.38MPa. However, it was more persistent in T+Night, with 1.78% observed 3 weeks after re-watering. The physiological mechanisms involved in tree mortality occur at different time scales (Anderegg *et al.*, 2012) and are linked to species-specific vulnerability to cavitation (Delzon *et al.*, 2010). In trees, Ψ_min_ is a relevant parameter to understand stem xylem cavitation and to define the thresholds of hydraulic failure (Brodribb *et al.*, 2010; Urli *et al.*, 2013). In the present findings, the minimum leaf water potential reached values close to –3MPa that probably induced xylem embolism (xylem air entry pressure, *P*_12_, being on average around –3MPa for this species). Moreover, in view of the standard deviation of both *P*_12_ and *P*_50_, it is possible that some individuals even reached their lethal cavitation threshold, explaining the mortality rate observed in the experiment. In sapling stems, the xylem tension inducing 50% loss of conductivity (*P*_50_) was –4.26MPa on average. Taken together, these results suggest that the saplings had a narrow safety margin under drought conditions and thus a high risk of hydraulic failure (Choat *et al.*, 2012). This result is in line with recent studies reporting that daily cycles of cavitation and successive repair are not habitual events for trees (Cochard and Delzon, 2013; Sperry, 2013; Wheeler *et al.*, 2013), as cavitation might only occur under severe drought (Delzon and Cochard, 2014). On the basis of the present results, it was considered that the hydraulic functionality of xylem would not be completely or irreversibly compromised for control saplings. However, the results were based on the response of a limited number of saplings, and dead individuals were not considered. In addition, the increase in mortality in non-irrigated saplings could be explained by a reduction in leaf hydraulic conductivity at warmer temperature. Indeed, during the post-drought period, saplings had dramatically lower rates of photosynthesis and stomatal conductance than those of pre-stress saplings and controls. This slow recovery phase might be due to a loss in leaf hydraulic conductivity associated with xylem cavitation. This could explain significantly the death of conifer saplings, as reported in Brodribb and Cochard (2009).

The higher mortality rate under warmer conditions could be due to temperature sensitivity when incomplete restoration of carbon reserves was reached, as observed in recent studies on conifers (Sala *et al.*, 2012; Adams *et al.*, 2013; Hartmann *et al.*, 2013). During water deficit, leaf parameters *g*_s_ and *A*_max_ declined to zero under all thermal conditions. A decrease in the maximum photosynthetic rate could normally be associated with a negative carbon gain, meaning that less sucrose would be translocated in the phloem and unloaded in cambium. However, NSC concentrations in both cambium and xylem were similar between the irrigated and non-irrigated saplings. The intra-annual pattern of NSC showed an inverse trend in the soluble sugar content between cambium (decline) and xylem (increase) around DOY 160 when starch in the xylem was near zero, suggesting the presence of strong seasonal dynamics, as observed in other conifers (Schaberg *et al.*, 2000; Gruber *et al.*, 2012). This seasonal scenario was often reported in spring and winter when starch to sugar conversion occurs (Schaberg *et al.*, 2000; Bucci *et al.*, 2003). The slower replenishment in starch reserves observed under warming, for both irrigated and non-irrigated saplings, could suggest an active role for starch, not only for the allocation of carbon resources for growth and metabolic demands, but also for the recovery of plants after drought.

It is hypothesized that the higher percentage of mortality at higher daytime and night-time temperatures, as well as the prolonged mortality at T+Night, could be related to the lower starch reserves after their seasonal minimum. The lower starch amount could be caused by a lower accumulation in the xylem due to a decrease in photosynthesis induced by water deficit. It is thought that the day and night daily fluctuation of starch in the stem could be analogous to that in the leaves. Thus, the lower recovery of starch in the xylem could reflect a change in the partitioning during the day and night. Reduction of carbon storage in the stem could be caused by (i) diminution of the fraction of carbon stored for later use or (ii) immediate use required to meet the higher metabolic demand at higher temperature. The co-occurrence of abiotic stresses thus limits the pools of stored carbon, possibly from lower sugar translocation by the phloem (Galiano *et al.*, 2011; Woodruff and Meinzer, 2011; Sala *et al.*, 2012). Recent research showed that under moderate drought, plant water conditions required for carbon remobilization sustained the survival of saplings, while severe drought strongly reduced the ability of saplings to utilize starch reserves, which did not ensure sapling survival (Hartmann *et al.*, 2013). Even if the present study is limited and precludes information on the NSC and starch pattern in other sink tissues (roots and leaves), the carbon starvation hypothesis cannot be invoked.

### Does the modification in wood anatomy make plants more resistant?

Under warmer conditions, xylem anatomy was modified by water deficit. Drought can induce the development of LW cells in EW, which is a typical reaction in species growing in the Mediterranean area (Cherubini *et al.*, 2003; de Luis *et al.*, 2011). In this study, however, at warmer temperatures, the observed plateau of cell wall thickness could represent the incapacity of black spruce to allocate sufficient carbon resource to build thicker cell walls. Moreover, a higher decrease (or lower plateau) was observed in non-irrigated saplings, clearly indicating a lower carbon allocation to cell wall development. The effect was also amplified as the water deficit occurred during the period of maximum cell production and differentiation. The co-occurrence of drought and warming that limited photosynthetic acclimation, with a consequent reduction in carbon (Way and Sage, 2008b), could influence the synthesis of cell wall components and produce thin cell walls (Luomala *et al.*, 2005). The most important consequence of such combined stress effects was the formation of wood with a lower density, which generally reflects a high hydraulic conductivity (Bucci *et al.*, 2004). This strategy does not allow the adaptation of black spruce toward a more efficient hydraulic system but probably decreases plant survival under warming and drought stress. Wood density is strongly correlated to drought-induced embolism (Pittermann *et al.*, 2006; Hoffmann *et al.*, 2011), because a low hydraulic conductivity may be an element of great drought resistance (Hacke *et al.*, 2001), but the relationship between wood density and resistance to cavitation is not direct. A lower wood density was recently proposed as a strategy to avoid catastrophic embolism after severe water deficit (Hoffmann *et al.*, 2011; Rosner *et al.*, 2014). The lower wood density could be caused by a change in the carbon allocation as (i) more carbon is required to meet the higher respiration demand at higher temperature, especially during the night (Amthor, 2000); and (ii) the carbon resources are mobilized for osmoregulation and are not available for cell wall building (Muller *et al.*, 2011; Pantin *et al.*, 2012).

## Conclusion

This experiment emphasizes the importance of investigating sapling responses to multifactor stress in order to reveal the effects on individual survival and xylem performance. The findings showed that the recovery of gas exchange never reached the initial pre-stress levels, indicating a loss in xylem hydraulic conductivity compared with pre-stress levels that could explain the hydraulic failure and death of individuals under warmer conditions. The consequences of drought under warming can improve our understanding of the role of wood density and carbon storage for sapling survival. This study underlined the importance of considering the active role of carbon storage and its utilization during tree growth under harsh environmental conditions. Although depletion of carbon reserves did not take place during prolonged water deficit, the carbon–water relationships changed and were important for the survival process in saplings.

## Supplementary data

Supplementary data are available at *JXB* online.

Figure S1. Sampling timetable of black spruce saplings.

Table S1. Means and *P-*values for total non-structural carbohydrates (NSCs) in cambium and in xylem and starch in xylem in black spruce saplings calculated between irrigation regimes and among thermal conditions, and interaction between irrigation regimes and thermal conditions

Supplementary Data

## Abbreviations:

*A*_max_: maximum photosynthesis rate
DOY: day of the year
EW: earlywood
*g*_s_: stomatal conductance
LW: latewood
*P*_12_: xylem air entry point
*P*_50_: pressure inducing 50% loss of hydraulic conductance
PLC_p_: predicted native embolism
T0: greenhouse with a temperature equal to the external air temperature
T+Day: greenhouse with temperature of 6 °C higher than T0 during the day
T+Night: greenhouse with temperature of 6 °C higher than T0 during the night
S: slope of the vulnerability curve of the stem
VC: vulnerability curve
VWC: volumetric water content of soil
WDp: water deficit period
Ψ_md_: midday leaf water potential
Ψ_min_: minimum leaf water potential
Ψ_pd_: pre-dawn leaf water potential.
