# Supplementary material for: How do drought and warming influence survival and wood traits of Picea mariana saplings?
Source: J Exp Bot. 2014 Nov 4;66(1):377–89. doi: 10.1093/jxb/eru431 (PMC4265170; doi:10.1093/jxb/eru431)
Supplement: Supplementary Data [file supp_66_1_377__index.html]

How do drought and warming influence survival and wood traits of Picea mariana saplings? — Supplementary Data 

# How do drought and warming influence survival and wood traits of *Picea mariana* saplings?

## Supplementary Data

Data files

**Files in this Data Supplement:**

- Supplementary Data - Supplementary Data
