# Supplementary material for: How do drought and warming influence survival and wood traits of Picea mariana saplings?
Source: J Exp Bot. 2014 Nov 4;66(1):377–89. doi: 10.1093/jxb/eru431 (PMC4265170; doi:10.1093/jxb/eru431)
Supplement: Supplementary Data [file supp_eru431_eru431_Supplementary_data_3june_REV.pdf]

Supplementary Figure S1 –. Sampling timetable of black spruce saplings. Black circles correspond to sampling days before, during and after the water deficit period (grey background). VWC correspond to volumetric water content of soil and NSCs correspond to non-structural carbohydrates.

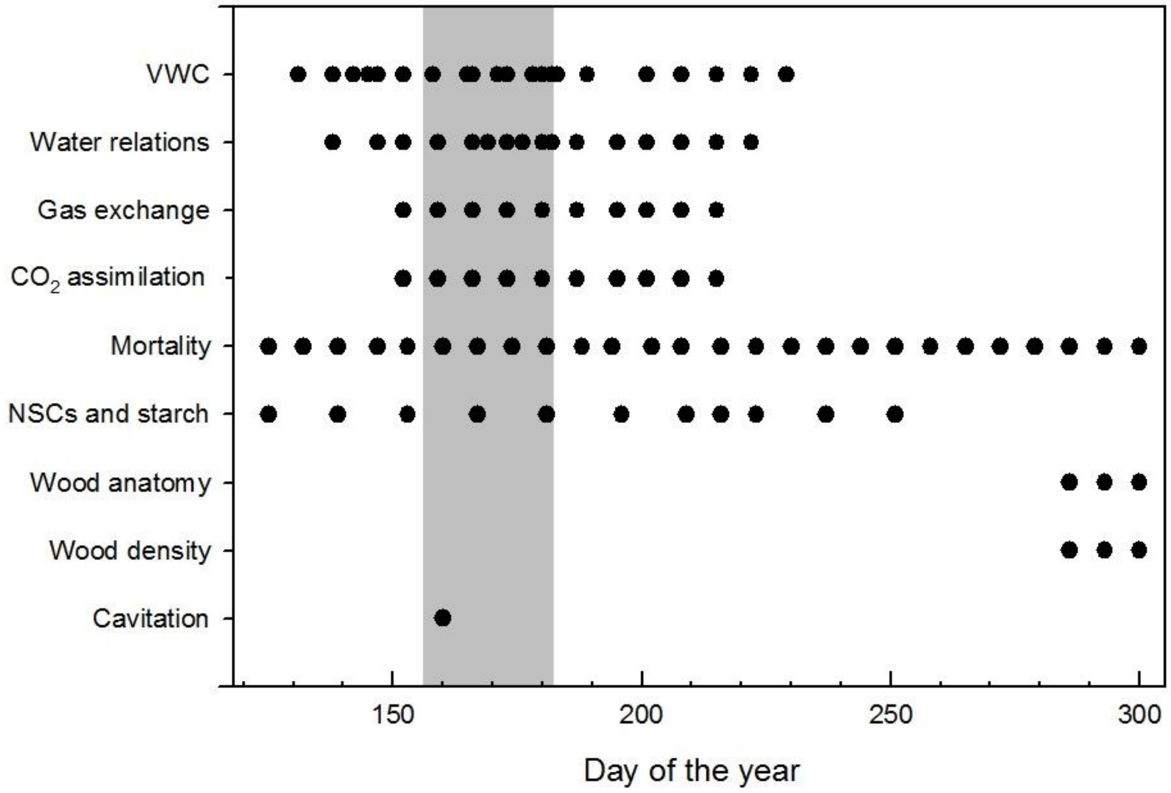

Supplementary Table S1

Means and *P* values for total non-structural carbohydrates (NSCs) in cambium and in xylem (mg/g_{dw}) and starch in xylem (mg/g_{dw}) in black spruce saplings calculated between irrigation regimes (I), among thermal conditions (T) and interaction between irrigation regimes and thermal conditions (I × T). Significant effects (*P≤ 0.05)* are in bold.

	**Irrigation regimes**						**Prob**			
	**Irrigated**			**Non-irrigated**			**I**	**T**	**I x T**	**DOY**
	**T0**	**T+Day**	**T+Night**	**T0**	**T+Day**	**T+Night**				
Total NSC in cambium	144.43	115.79	138.73	135.63	137.75	139.18	0.44	0.05	0.05	**<0.001**
Total NSC in xylem	9.21	8.67	8.90	8.79	9.27	8.87	0.90	0.96	0.54	**<0.001**
Starch in xylem	4.17	3.09	3.09	4.46	2.71	3.42	0.14	**<0.001**	0.67	**<0.001**
